# The Payment of Benefits

**Published:** 1913-01-18

**Authors:** 


### OUR NATIONAL INSURANCE ACT DEPARTMENT.

### XXXIX.—The Payment of Benefits.

The National Insurance Act has now entered upon another stage in its progress, as sickness and maternity benefits commenced to become payable on Monday last, and medical benefit came into operation on Wednesday. It is, of course, too early to say whether the payments of the benefits will produce the effect of rendering the Act popular throughout the country, as has been claimed by its most ardent supporters. If the payment of benefits does not ultimately produce this effect the measure will have proved a failure. Popular favour must, after all, be the final test. A great deal will depend upon the way in which the benefits are paid. Delay and red-tape inquiries will lead to irritation, and it cannot be too strongly impressed upon the officers of the approved societies as well as upon the officials of the Insurance Commissions that promptness in paying claims is absolutely essential. It is largely due to the businesslike arrangements made by the large industrial assurance companies in the matter of paying claims that they have attained such phenomenal success, and have expanded so rapidly in recent years.

The delay in making the arrangements for the administration of medical benefit unfortunately made it well-nigh impossible for the approved societies to obtain official information as to the precise wording of the medical certificates that are to be used when claiming sickness benefit, and in other indirect ways hindrances have occurred which must have some effect on the facility for paying claims during the first few weeks. Three months at the outside should, however, be sufficient time for all the officials concerned to become familiar with their ordinary duties, and after the expiration of that time it will be possible to judge whether the complicated rules and regulations are capable of being carried out in practice, or whether they will have to be simplified in any marked degree.

### The Provision of Funds.

A very disquieting report was printed very generally in the daily newspapers at the beginning of last week to the effect that a notice had been sent to the approved societies calling upon them to pay the benefits out of their private funds, and then going on to point out that repayments would be made by the Insurance Commissioners by monthly accounts for disbursements actually made, subject to a deduction of 10 per cent. until the accounts have been audited. Such a statement naturally met with severe criticism, for the so-called waiting period of six months was expressly provided in order that there should be sufficient funds in hand to meet the payment of benefits when they began to accrue. It would, therefore, have been unjust to make any such requirement as that indicated. Such a wide circulation was given to the report—generally under display type, calling attention to the fact that the friendly societies had to finance the Act—that a rejoinder was immediately given by the Insurance Commissioners pointing out that the report was without foundation in the matter of its general applicability. As so often occurs in similar circumstances, the denial was not given the same prominence as the original statement, and we observed in a paper bearing Saturday's date, or some three days after the contradiction had appeared, a notice in similar terms to the original report, without any note of the correction. In the circumstances it appears desirable to state precisely the actual position. This may be done somewhat as follows.

### The Position Explained.

The Act contains provisions the object of which is to protect insured persons against loss arising from bad management or malversation on the part of the societies' officials. At the outset the societies themselves had to be approved—that is to say, their rules and constitution had to fulfil the requirements of the Commissioners. A constitution on paper and a book of rules does not necessarily protect from loss, even when almost every act is closely watched by Government officials. It was therefore necessary to make provision in the Act (Section 26) that every society desirous of being approved should furnish such security to the Insurance Commissioners as the latter may deem appropriate in regard to the magnitude of the interests affected, to provide against any malversation or misappropriation by officers of the society of the society's funds coming into their hands. In other words, a guarantee policy was required as a condition of approval, but to this there was one important exception, as it was quite rightly provided that when a society could prove to the satisfaction of the Commissioners that the only funds coming into the hands of the officials would be in the nature of a reimbursement for sums already expended no such security should be required. In making the necessary arrangements for the provision of funds the Commissioners sent a form to each society requesting to be informed on a specially franked postcard which method the society intended to adopt. By some mischance, at present unexplained, particulars relating to the system of provision of funds by reimbursement were sent to a society that had wished to obtain its funds from the Commissioners under the plan of guarantee. Thus the report was issued to the press under conditions that were never intended to apply, and it is a relief to be assured that there will be no failure of funds to meet the payments as they become due. It was stated in Parliament a few days ago that the sum received on account of National Health Insurance stamps up to the end of last year was over £8,000,000, thus making with the proportion of benefits payable by Government a sum of approximately £10,000,000 available for the payment of benefits.

Last week we gave in outline the conditions that have to be fulfilled before an insured person is entitled to sickness or maternity benefits. It may be as well to supplement that statement by pointing out that conditions not only have to be fulfilled before the benefits can become payable, but in the case of sickness benefit further conditions have to be complied with in order that the member may be entitled to continue in receipt of the benefit. These conditions are not necessarily identical in all societies, though their general purport will be similar. The conditions are contained in the rules of the societies and are generally known as rules relating to conduct whilst in receipt of sick-pay.

The rules are framed with the object of protecting the society, and thus the bulk of the members, from the imposition of the few who without such restrictions might be tempted to malinger. Thus a common form of rule provides that no member may return to work without sending to the secretary not later than the same day notice declaring off the fund. It is also a common form of rule to require that a member shall not do any work whilst in the receipt of benefit, and, further, that he shall be required to submit to medical examination as and when required should the society have any reason to demand a report other than the usual weekly reports of the doctor or sick visitor in attendance. A still further rule that in some shape or form is almost certain to appear amongst those of all societies deals with the absence of the insured person from home. The same hours are not fixed in all cases, but it is by no means unusual to find it stated that a member in receipt of benefit may not be absent from home between the hours of 7 P.M. and 7 A.M. without special permission having been accorded, and in no event may leave home without letting it be known where he may be found. Members of friendly societies are, of course, perfectly familiar with these rules, but the majority of insured persons have not the same acquaintance with them, and it is quite likely that many persons have joined societies without having taken the trouble to read the rules or to understand the conditions of membership. We strongly advise our readers who may be affected not to leave the matter to chance, but to make a point of reading the rules of the societies to which they may belong. Various penalties may be imposed for breach of rules, and these are recognised by the Act, which, in Section 14, specifically defines the limits of the penalties that may be imposed. Briefly, a fine not exceeding 10s. may be imposed, or in the case of repeated breach of rules it may be as much as £1 or suspension from benefit for a period not exceeding a year. There are circumstances also under which it may be possible to expel a member as, for instance, when it can be proved that membership was obtained by means of false and fraudulent statements on his part.

It is sufficient to point out here that the Act contemplates that everything shall be done decently and in order, and for that reason alone it is advisable for all members of approved societies to read and endeavour to understand the rules of the societies to which they belong.

Medical and Surgical Appliances.

Some weeks ago an inquiry was addressed to our Bureau of Information asking whether an artificial limb would come within the description of the surgical appliances that are promised under the heading of medical benefit by the National Insurance Act. At that time we were unable to reply definitely as the Regulations prescribing the medical and surgical appliances that are to be supplied had not then been published. We indicated, however, that we thought it extremely unlikely that any such appliances as artificial limbs would be provided, because the cost would be too great for the insurance funds to bear. There are, doubtless, a large number of our readers, beside the correspondent referred to, to whom the matter is of interest, and it may be as well to furnish the list of appliances now published in the statutory Regulations made by the Insurance Commissioners for the administration of medical benefit.

The list is given in the second schedule of the Regulations and is as follows:—Bandages, gauzes, lints, wools, oiled silk, oiled paper, gutta-percha tissue, adhesive plaster, ice-bags, splints, and catheters. As actually printed the list appears to be at least twice the length of that just given, for in order to make the Regulations precise a description of the various kinds of bandages, gauzes, lints, and wools that will be supplied is given. Thus, for example, the bandages may be either bleached or unbleached calico, crêpé, domette, flannel, india-rubber, muslin, plaster of Paris, or open-wove.

It is thus clear that we were correct in our assumption as to the provision of artificial limbs, and it is apparent that only such medical and surgical appliances are to be supplied as come within the meaning attached to medical benefit by the Insurance Commissioners—namely, such treatment as can be given by a practitioner of ordinary professional competence and skill.

A list of drugs that may be prescribed by practitioners in attendance on insured persons is also to be drawn up, and will be in the hands of doctors and chemists taking service under the Act. If necessary, drugs other than those included in the list may be ordered by the doctor, but if he does so the order is to be made on a special form. Chemists taking service under the Act are required by the statutory Regulations, so far as practicable, to keep in stock the drugs and appliances specified, and to supply them with reasonable promptness to any person presenting an order signed by any practitioner on the panel.

